# Phase II pilot study of the prednisone to dexamethasone switch in metastatic castration-resistant prostate cancer (mCRPC) patients with limited progression on abiraterone plus prednisone (SWITCH study)

**DOI:** 10.1038/s41416-018-0123-9

**Published:** 2018-08-21

**Authors:** Nuria Romero-Laorden, Rebeca Lozano, Anuradha Jayaram, Fernando López-Campos, Maria I. Saez, Alvaro Montesa, Ana Gutierrez-Pecharoman, Rosa Villatoro, Bernardo Herrera, Raquel Correa, Adriana Rosero, María I. Pacheco, Teresa Garcés, Ylenia Cendón, Ma Paz Nombela, Floortje Van de Poll, Gala Grau, Leticia Rivera, Pedro P. López, Juan-Jesús Cruz, David Lorente, Gerhardt Attard, Elena Castro, David Olmos

**Affiliations:** 1 Prostate Cancer Clinical Research Unit, Spanish National Cancer Research Centre (CNIO), Madrid, Spain; 2 Medical Oncology Department, Hospital Universitario de La Princesa, Madrid, Spain; 3 Medical Oncology Department, Hospital Universitario de Salamanca, Salamanca, Spain; 4 CNIO-IBIMA Genitourinary Cancer Research Unit, Institute of Biomedical Research in Málaga (IBIMA), Málaga, Spain; 5 Division of Molecular Pathology, Centre for Evolution and Cancer, The Institute of Cancer Research, London, United Kingdom; 6 Academic Urology, The Royal Marsden NHS Foundation Trust, London, United Kingdom; 7 Radiation Oncology Department, Hospital Universitario Ramón y Cajal, Madrid, Spain; 8 Medical Oncology Department, Hospitales Universitarios Virgen de la Victoria y Regional de Málaga, Málaga, Spain; 9 Pathology Department, Hospital Universitario de Móstoles, Móstoles, Spain; 10 Medical Oncology Department, Hospital Costa del Sol, Marbella, Spain; 11 Urology Department, Hospital Universitario Virgen de la Victoria, Málaga, Spain; 12 Radiation Oncology Department, Hospital Universitario Virgen de la Victoria, Málaga, Spain; 13 Oncology Department, Hospital Universitario Fundación Alcorcón, Alcorcón, Spain; 14 School of Medicine, Universidad Autónoma de Madrid, Madrid, Spain; 15 Medical Oncology Department, Hospital Universitario La Fe, Valencia, Spain; 16 Medical Oncology Department, Hospital Universitario Quirón, Madrid, Spain

**Keywords:** Prostate cancer, Cancer therapeutic resistance

## Abstract

**Background:**

Despite most metastatic castration-resistant prostate cancer (mCRPC) patients benefit from abiraterone acetate plus prednisone 5 mg bid (AA + P), resistance eventually occurs. Long-term use of prednisone has been suggested as one of the mechanisms driving resistance, which may be reversed by switching to another steroid.

**Methods:**

SWITCH was a single-arm, open-label, single-stage phase II study. The primary objective was to evaluate the antitumour activity of abiraterone acetate plus dexamethasone 0.5 mg daily (AA + D) in mCRPC patients progressing to AA + P. Clinically stable mCRPC patients who had prostate-specific antigen (PSA) and/or limited radiographic progression after at least 12 weeks on AA + P, were eligible. The primary endpoint was measured as the proportion of patients achieving a PSA decline of  ≥ 30% (PSA30) from baseline after 6 weeks on AA + D. Secondary endpoints included: PSA50 response rate at 12 weeks, time to biochemical and radiological progression, overall survival, safety profile evaluation, benefit from subsequent treatment lines and the identification of biomarkers of response (*AR* copy number, *TMPRSS2-ERG* status and PTEN expression).

**Results:**

Twenty-six patients were enrolled. PSA30 and PSA50 were 46.2% and 34.6%, respectively. Median time to biochemical and radiological progression were 5.3 and 11.8 months, respectively. Two radiological responses were observed. Median overall survival was 20.9 months. Patients with *AR* gain detected in plasma circulating tumour DNA did not respond to switch, whereas patients with *AR* normal status benefited the most. No significant toxicities were observed and PSA50 response rate to subsequent taxane was 50%.

**Conclusions:**

In selected clinical stable mCRPC patients with limited disease progression on AA + P, a steroid switch from prednisone to dexamethasone can lead to PSA and radiological responses.

## Introduction

Although novel therapeutic options are being developed for metastatic castration-resistant prostate cancer (mCRPC), new rational-based strategies may optimise the benefit from currently available therapies such as abiraterone acetate (AA)^1,2^.

AA inhibits androgen synthesis through blockade of CYP17 17α-hidroxylase and 17,20-lyase functions. Continuous CYP17 inhibition may result in rising adrenocorticotropic hormone levels and increased steroid levels upstream of CYP17, which may not only prevent adrenocortical insufficiency but also could result in a secondary mineralcorticoid excess characterised by fluid retention, hypertension and/or hypokalemia^3^. To prevent these, AA is administered in combination with prednisone 5 mg twice daily^1,2^.

Nonetheless, prednisone has not been the only concomitant steroid used with AA. In the initial phase I/II trial, AA was administered without steroids, and dexamethasone 0.5 mg od was only added to single AA after biochemical progression^4,5^. This strategy led to a prostate-specific antigen (PSA) decline > 50% (PSA50) in 33% of patients, suggesting a reversal of resistance to AA^4^. In the first reported post-docetaxel phase II trial of AA, Reid et al.^6^ also used dexamethasone 0.5 mg od. This study showed a PSA50 response rate of 51%, whereas a contemporaneous phase II trial with AA plus prednisone 5 mg bid observed a PSA50 response rate of 39%^7^.

A potential difference in the activity of AA in combination with prednisone and dexamethasone may also be supported by the superiority of dexamethasone in monotherapy over prednisone in terms of PSA response (47% vs 24%, *p* = 0.05) and median time to PSA progression (9.7 vs 5.1 months) as demonstrated in a randomised phase II trial in mCRPC patients^8^. In this study, crossover to dexamethasone in patients progressing to prednisone was associated with 37% biochemical responses^8^.

The hypothesis that the switch of prednisone to dexamethasone in patients with biochemical progression to AA plus prednisone would achieve secondary responses has been explored in a retrospective post-docetaxel cohort. Biochemical responses were observed in 40% of the cases included in this series^9^. Here, we present the data of a prospective phase II study of mCRPC patients treated with AA 1000 mg od plus dexamethasone 0.5 mg od (AA + D) after biochemical and/or limited radiographic progression to AA 1000 mg od plus prednisone 5 mg bid (AA + P) pre- and post docetaxel.

## Materials and methods

### Patient population

The SWITCH study (NCT02928432) was a prospective multicentre study conducted at four university hospitals in Spain. Castrate (serum testosterone ≤ 50 ng/dL) metastatic prostate cancer patients with Eastern Cooperative Oncology Group (ECOG) performance status 0–2 who had a histological diagnosis of prostate adenocarcinoma, a PSA > 2 ng/mL, and confirmed biochemical progression as defined by PCWG2 criteria^10^ after at least 12 weeks of AA 1000 mg od and prednisone 5 mg bid were eligible. Patients with limited radiological progression on AA+P (as defined by: i.  ≤ 3 new asymptomatic metastasis in bone scan, ii. no new soft tissue lesions, and iii. < 40% increase in the size of target lesions according to RECIST1.1^11^) were allowed in the study. Patients had to be asymptomatic or present stable symptoms without any worsening in grade. A complete list of eligibility criteria is provided as Supplementary Appendix. Eligible patients were enrolled in the study after providing informed consent. The study was approved by the institutional ethics review committees of all participating centres and was conducted in accordance with the Declaration of Helsinki and International Conference on Harmonisation/WHO Good Clinical Practice standards.

### Study design and response assessment

This was a single-arm, open-label, single-stage phase II study. The primary objective was to evaluate the antitumour activity of AA+D in patients with mCRPC who had biochemical progression (with or without limited radiological progression as described above). The primary endpoint was measured as the proportion of patients achieving a PSA decline of ≥ 30% (PSA30) from baseline after 6 weeks on AA+D, and confirmed by a second PSA value ≥ 2-weeks later. The proportion of patients achieving PSA50 response after ≥ 12 weeks on AA+D was also reported as secondary endpoint as per PCWG2 recommendation. Time to PSA progression was defined as the date that a ≥ 25% increase in PSA with an absolute increase of ≥ 2 ng/mL above the nadir occurred. Confirmation by a second PSA value ≥ 2-weeks later was required. Measurable disease response rate using RECIST1.1 and PCWG2 criteria was assessed at least 12 weeks after AA+D initiation. Patients with biochemical progression were allowed to continue on AA+D until radiographic or clinical progression, whichever occurred first.

### Treatment and procedures

Four tablets (250 mg each) of AA and one capsule (0.5 mg) of dexamethasone were administered daily, continuously, in 28-day cycles. All patients underwent a standard evaluation that included prior medical history, physical examination, and laboratory tests (PSA, haematology, biochemistry, liver and renal function studies) at baseline and at 2-weeks intervals for the first 8 weeks and then in 4 weeks intervals. AEs were graded according to the National Cancer Institute Common Toxicity Criteria for Adverse Events, version 4.0. Baseline high-resolution computed tomography scans and bone-scans were performed and repeated every 12 weeks.

### Biomarker studies

Available archival prostate cancer formalin-fixed paraffin-embedded samples and optional plasma samples at progression to abiraterone and prednisone within 4 weeks prior to first dose of dexamethasone were collected. PTEN protein expression was determined by immunohistochemistry as previously described^12^. *TMPRSS2-ERG* fusion was assessed by fluorescent in-situ hybridisation using a modified three-colour assay based on the *ERG* break-apart assay described by Attard et al.^13^. Plasma was obtained by centrifugation of 10 mL of blood collected in ethylenediaminetetraacetic acid tubes within 2 hours from blood-drawn and stored at −80 °C. DNA was extracted from 2 ml of plasma and *AR* status in plasma was determined by digital drop PCR (ddPCR) using the QX200 ddPCR system (Bio-Rad), for *AR* copy number as described previously^14^, and for *AR* mutations (supplementary appendix S2).

### Statistical analyses

The primary aim of the study was to demonstrate the rate of patients that showed a ≥ 30% decline in PSA at 6 weeks. A single-stage A’Hern phase II trial design^15^ was used to estimate the sample size. By using a response rate of 10% for the null hypothesis versus an alternative hypothesis response rate of 30%, an alpha-error of 0.05 and a power of 0.80, 25 patients were to be recruited. The null hypothesis would be rejected if at least six patients had a PSA decline ≥ 30%. Biomarker studies were exploratory and descriptive statistics were used.

## Results

### Patient characteristics

Twenty-six mCRPC patients were enrolled in this Phase II trial between June 2013 and March 2016 (CONSORT diagram supplementary appendix S3). Median age was 72.6 years (range 60.2–85.8), median PSA 36.1 ng/mL (range 4.5–1880) and 96% were ECOG 0–1. Excluding AA + P, the median number of prior treatment lines for mCRPC was 1 (range 0–3). Fourteen patients (53.8%) were chemotherapy-naive at the initiation of AA+P. Median time on AA + P was 6.2 months (range 3.0–31.3). All patients had PSA progression and 12 (46.2%), also presented a limited radiological progression as predefined in the study inclusion criteria. Their clinical characteristics are summarised in Table 1. Median time on AA + D was 8.6 months (range 1.8–28.5 months). At the time of data cutoff (30 May 2017) the three patients that remained on treatment had been on AA + D for 15.2, 15.4 and 19.5 months, respectively.

**Table 1:** Baseline characteristics of 26 patients included in the study

Characteristics	All (*n*=26)		AA + D pre-chemo (*n*=14)		AA + D post chemo (*n*=12)	
	*N*	%	*N*	%	*N*	%
Age						
Median (range)	73.0 years	(60–85)	72.5 years	(60–85)	73 years	(66–78)
Baseline PSA						
Median (range)	36.1	(4.46–965.2)	20.6 ng/mL	(4.5–367.0)	39.9 ng/mL	(6.9–1880)
Gleason						
6	4	15%	2	14%	2	17%
7	7	27%	3	21%	4	33%
8–10	14	54%	8	57%	6	50%
ECOG						
0	10	38%	6	43%	4	33%
1	15	58%	7	50%	8	67%
2	1	4%	1	7%	–	–
Time to CRPC						
Median (range)	24.3 months	(6.2–145.1)	23.3 months	(6.2–145.1)	34.8 months	(19.1–107.5)
Previous steroids to AA						
Monotherapy	3	12%	3	21%	–	–
Docetaxel	12	46%	–	–	12	100%
Cabazitaxel	1	4%	–	–	1	8%
> 6 months	11	42%	2	14%	9	75%
< 6 months	4	15%	1	7%	3	25%
Metastasis						
Bone	24	92%	12	86%	12	100%
Nodes	12	46%	8	57%	4	33%
Visceral	4	15%	1	7%	3	25%
Progression to AA + PRED						
Biochemical (PSA)	26	100%	14	100%	12	100%
Radiological (new)§	8	31%	4	29%	4	33%
Radiological (size)	4	15%	2	14%	2	17%
LDH						
Normal	14	54%	8	57%	6	50%
High	12	46%	6	43%	6	50%
Alkaline phosphatase						
Normal	19	73%	4	29%	9	75%
High	7	27%	10	71%	3	25%
Haemoglobine						
> 10 g/dL	23	88%	13	93%	10	83%
≤ 10 g/dL	3	12%	1	7%	2	17%
Albumin						
≥ 35 g/L	19	73%	11	79%	8	67%
< 35 g/L	5	19%	2	14%	3	25%
AA + PRED cycles (28 days)						
Median (range)	6.2	(3.0–31.3)	5.8	(3.0–28.1)	6.4	(3.0–31.3)
PSA response to AA + PRED						
PSA decrease ≥ 50%	12	46%	6	43%	6	50%
PSA decrease ≥ 30% and < 50%	1	4%	–	–	1	8%
No response	13	50%	8	57%	5	42%

§Three new bone metastasis in bone scan and/or an increase of target lesions <40%. *AA* Abiraterone acetate, *P* prednisone, *D* dexamethasone

### Antitumour activity

**Fig. 1:**
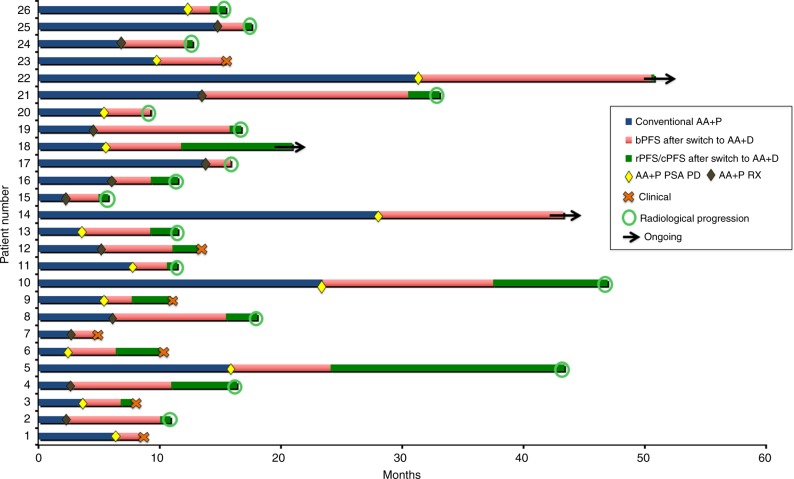
Swim lanes illustrating the 26 patients on the trial. Blue bars represent time on abiraterone plus prednisone. Diamonds represent progression to abiraterone plus prednisone (biochemical in yellow, biochemical+radiological in brown) and starting date on AA+D (switch). Red bars represent time to biochemical progression after switch. Green bars represent time between biochemical progression and radiological or clinical progression-free survival. AA+P, abiraterone plus prednisone; bPFS, biochemical progression-free survival; rPFS, radiolographic progression-free survival; cPFS, clinical progression-free survival; PSA PD, biochemical progression; RX PD, radiological progression

**Fig. 2:**
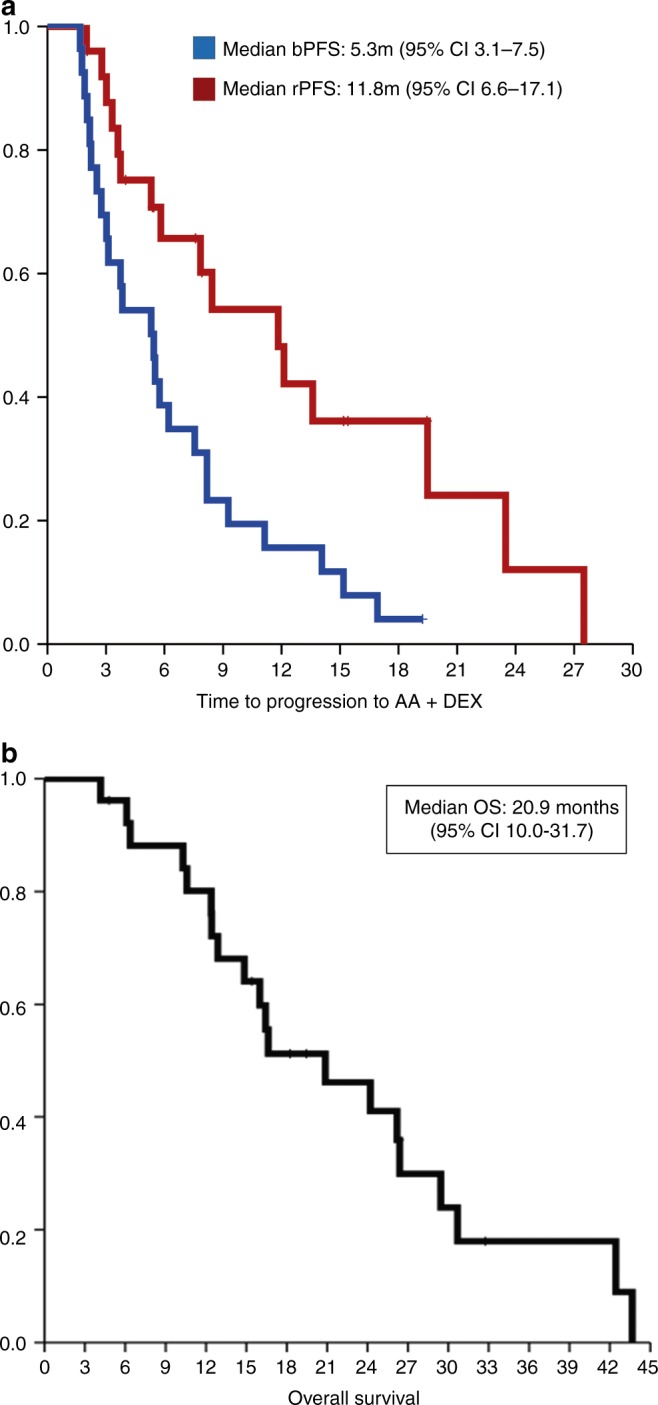
Kaplan–Meier survival curves. **a** biochemical (PSA) progression-free survival (bPFS) and radiographic progression-free survival (rPFS) survival curves are represented in blue and in red, respectively. **b** Overall Survival (OS)

The swimmer-plot in Fig. 1 summarises the experience of patients on AA+P and AA+D. Following at least 6 weeks from AA+D switch 12 patients (46.2%) presented a PSA30 response. PSA50 response rate at ≥ 12 weeks was 34.6% (*n* = 8). PSA50 response to AA+D was observed in 5 out 12 (41.7) and 3 out of 14 (21.4%) patients with or without previous PSA50 response on AA+P, respectively. In patients with or without prior docetaxel, PSA50 response rates were 41.7%, and 21.4%, respectively. In this series, median biochemical progression-free survival (bPFS) with AA+P was 5.4 months. Median bPFS from AA+D switch was 5.3 months (95% CI 3.1–7.5) Fig. 2a. There was a moderate (*r* = 0.5) but significant correlation (*p* = 0.001) between bPFS on AA+D and bPFS on AA+P.

**Fig. 3:**
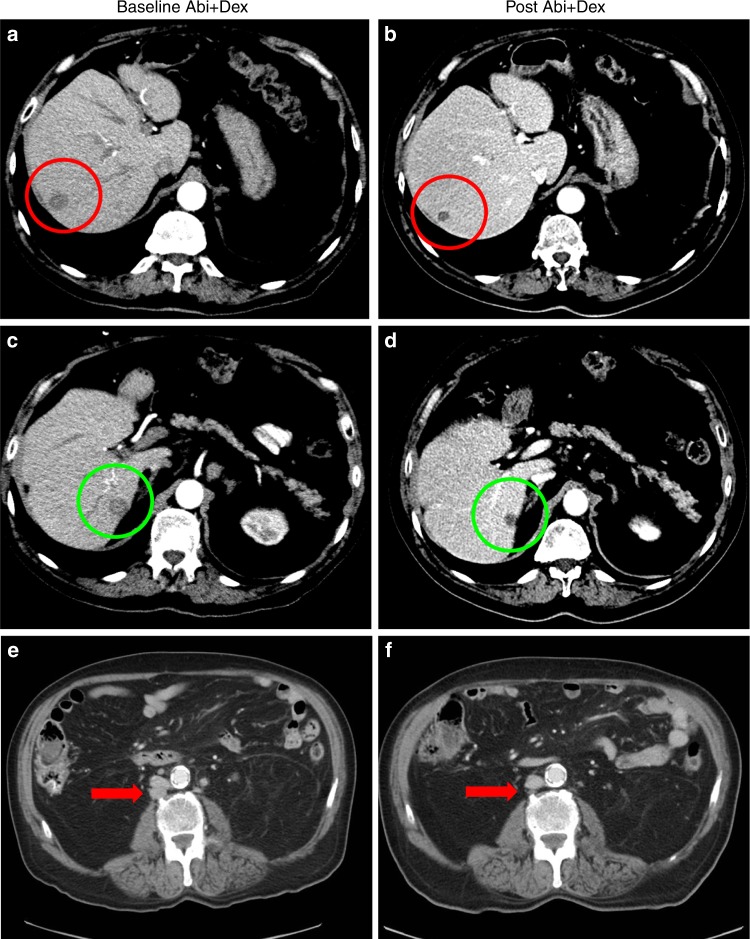
**a**–**d** illustrate the response of Patient 004, who had previously experienced progression on a luteinizing hormone-releasing hormone agonist (LHRHa) and bicalutamide. After 16 weeks on AA+P he presented with confirmed biochemical and radiological progression of liver metastases (30% increase of target lesions) **a** and **c**. Then, patient was switched from concomitant prednisone 5 mg bid to dexamethasone 0.5 mg od. After 12 weeks on AA+D, PSA decreased to a nadir of 0.61 ng/mL (88% decline), and **b** and **d** the red and green oval, respectively, show a partial response of his target liver metastases. Patient 004 continued on AA+D until clinical and radiographic progression occurred 13.6 months after the switch. **e**, **f** show the response of patient 022. This patient had previously been treated with LHRHa, bicalutamide, ketoconazole/hydrocortisone, and docetaxel/prednisone. Patient responded to AA+P. After 14.8 and 31.3 months he experienced biochemical and radiological progression, respectively. **e**. Patient 022 was then switched to dexamtehasone and reached a biochemical response ( > 90%) and a significant radiological response of target nodal disease **f**, red arrow) still ongoing after 19.2 months on the study at the time of data cut–off. Patients 004 and 022 did not presented AR amplification or detectable mutations in ctDNA at time of switch

Median time to radiographic progression (rPFS) after the switch was 11.8 months (95% CI 6.6–17.1), Fig. 2a. In the pre- and post-docetaxel settings median rPFS was 13.6 and 11.8 months, respectively. According to RECIST1.1 criteria, two objective partial responses were observed in a patient with liver metastasis (Fig. 3a–d) and in a second patient with measurable nodal disease (Fig. 3e, f).

### Overall survival and effect of SWITCH on subsequent therapies

Median OS since AA+D initiation was 20.9 months (95%CI 10.0–31.7), Fig. 2b. Effect of subsequent therapies for mCRPC was evaluated in 20 out of 23 patients with clinical and/or radiological progression who had started a new treatment line after AA+D at the time of data collection cutoff. Docetaxel (40%), Ra-223 (30%) and enzalutamide (15%) were the most-frequent subsequent-line after AA+D, see supplementary appendix S5. Twelve patients received at least 1 taxane (11 docetaxel, 1 cabazitaxel) as first-chemotherapy following AA+D (9 immediately after AA+D, 2 after Ra-223, and 1 after enzalutamide). PSA50 response rate to taxanes at ≥ 12 weeks in these 12 patients was 50%.

### Safety evaluation

Eight patients (31%) presented at least one grade 1–2 related adverse events (AEs) after switch to AA + D. No grade 3–4 related AEs were reported. The commonest AA + D related AEs were muscle weakness (*n* = 3, 12%), hypertension (*n* = 2, 8%) and hyperglycaemia (*n* = 2, 8%). An episode of orthostatic hypotension without other symptoms of adrenocortical insufficiency during a concurrent episode of acute gastroenteritis was reported as possibly related to AA+D. Prior study enrolment, three patients on AA+P had been started on eplerenone 25–50 mg od due to mineralcorticoid excess syndrome (oedema, hypertension and/or hypokaliemia). A fourth patient was started on oral antidiabetics due to AA+P related hyperglycaemia. These side-effects were controlled in all the four patients at time of switch to AA+D and did not require treatment adjustment, whereas on dexamethasone. Related AEs on study are summarised in Table 2.

**Table 2:** Treatment-related adverse events

	AA + Prednisone			AA + Dexamethasone		
	Grade 1	Grade 2	Grade 3/4	Grade 1	Grade 2	Grade 3/4
Hypertension	1	2	0	1	1	0
Hypokalaemia	2	0	0	0	0	0
Oedaema	1	0	0	0	0	0
Hyperglycaemia	1	0	0	1	1	0
Hypertransaminasemia	1	0	0	0	0	0
Hypotension	0	0	0	1	0	0
Muscle weakness	0	0	0	3	0	0
Total events	6	2	0	6	2	0

### Biomarker studies

**Fig. 4:**
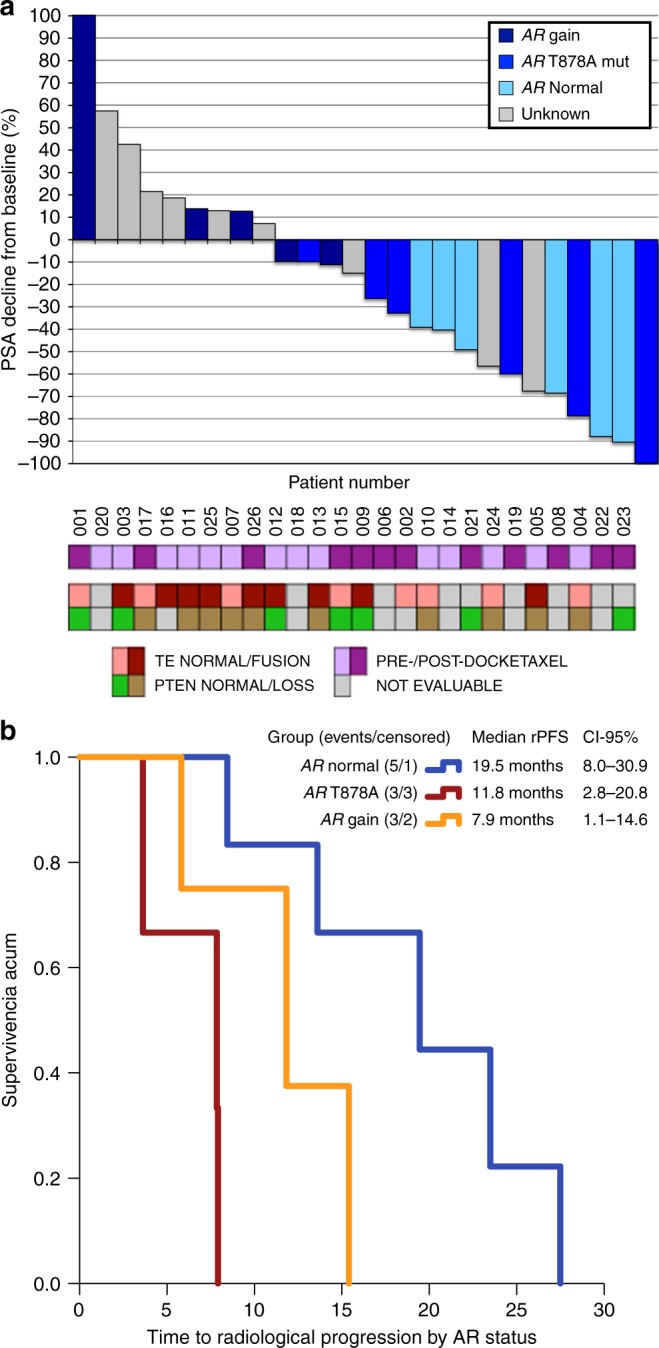
**a** Waterfall plot representing PSA best response according to PCWG2 criteria (*y*-axis) and patients (*x*-axis). Each individual bar represents a patient, ordered by the magnitude of PSA response. Patients’ ID at the bottom match those in Fig. 1 (swimmer-plot). Each bar is coloured according to *AR* status determined in ctDNA: navy-blue for *AR* gain, medium-blue for *AR* T878A mutation, sky-blue for *AR* normal, grey for unknown status due to lack of sample available, or low cfDNA isolated. Panel at the bottom summarises pre- or post-docetaxel status, *TMPRSS2-ERG* (TE) fusion and PTEN status in available archived diagnostic biopsies/prostatectomy. **b** Kaplan–Meier radiographic progression-free survival (rPFS) curves according to *AR* status in ctDNA: blue represents patients with *AR* normal status, orange for patients harbouring an *AR* T878A mutation, and red for patients with *AR* gain detected, respectively. Exploratory long rank-test suggests that rPFS is significantly prolonged in *AR* normal compared with *AR* gain (*p* = 0.002). The differences observed between *AR* normal and *AR* T878A (*p* = 0.117) or *AR* T878A and *AR* gain (*p* = 0.092) were not significant

*AR* copy number, T877A and L702H mutation status in plasma ctDNA at AA + D baseline, as well as PTEN and *ERG* rearrangement status in tissue were determined in patients with available samples (supplementary appendix S6). The best PSA change at any time after 12 weeks according to *AR* status in plasma ctDNA and other biomarkers is presented in Fig. 4a. PSA30 and PSA50 response rates in patients with *AR* normal were 100% and 50%, respectively. None of the five patients with *AR* gain had a PSA response and compared with *AR* normal patients showed significantly shorter bPFS (2.8 vs 8.3 months, *p* = 0.001) and rPFS (7.9 vs 19.5 months, *p* = 0.002), Fig. 4b. *AR* T878A mutation was detected in six patients at switch, PSA30 and PSA50 response rates were 67% and 50%, respectively. Median bPFS and rPFS for this group were 5.3 and 11.8 months, respectively, which did not differ significantly from *AR* gain. *TMPRSS2-ERG* rearrangement was present in 9 (52.9%) patients. PSA30 response rates were 11.1% and 50% in patients with or without *ERG* rearrangement, respectively, but not significant differences in bPFS or rPFS were seen.

## Discussion

The SWITCH trial was a proof of concept study that, for the first time, prospectively evaluated the antitumour activity of a steroid switch from prednisone 5 mg bid to dexamethasone 0.5 mg od concomitant to AA 1000 mg od. We report durable PSA declines, two objective radiological responses and several prolonged disease stabilisations in clinically stable patients progressing to AA + P. This extension of time on therapy associated to a clinical benefit is a meaningful therapeutic objective^16^. Activity was seen in both pre- and post-docetaxel settings. Importantly, AA + D switch in this study did not add any significant toxicities to AA+P and did not compromise subsequent treatment with taxanes.

Our study met its primary endpoint by proving that the steroid switch could induce a PSA decrease ≥ 30% in at > 30% of patients (46.2%). A PSA30 response rate at 6 weeks was chosen as a primary endpoint in the trial in order to minimise the patients’ exposition to a potentially ineffective strategy while maximising the possibility of identifying significant antitumour activity. Despite the fact, this is not a standard definition as per PCWG criteria^10,17^, recent analyses support the potential utility of a PSA30 response rate endpoint: PSA30 has been associated with improved survival in patients treated with taxanes^18^ and abiraterone^19^. On the other hand, PSA50 response rates at 12 weeks were concordant with PSA30 responses at 6 weeks, further supporting our alternative hypothesis.

Current guidelines recommend the maintenance of AA + P beyond PSA rise until clinical and/or radiological progression ocurrs^20^. In our study, all patients experienced PSA progression to AA+P, and approximately half of them (14/26) presented radiographic progression. Median rPFS from the AA + D switch was, approximately, 11.8 months, which is longer than the median 5.4 months that was observed from biochemical to radiographic progression in the abiraterone arm of the COU-302 trial^2^. Our results also compare favourably (rPFS 11.8 months, PSA response: 34.6%) to a recently presented phase IV study in which a highly selected population of patients who had disease progression after ≥ 24 weeks on treatment with AA + P received enzalutamide. In this study, median rPFS was 8.1 months with an unconfirmed PSA response rate of 27%^21^. Furthermore, switching steroids on progression to AA + P was recognised as a valid therapeutic option in the recent St. Gallen Advanced Prostate Cancer Consensus Conference, where 72% of panellists recommended a steroid switch in selected patients^22^ despite the lack of prospective evidence available at the time and which we provide for the first time.

Noteworthy, time to bPFS and rPFS were shorter in patients with *AR* gain compared with *AR* normal. In addition, none of the patients with *AR* gain responded by PSA to AA + D, whereas all *AR* normal presented a decline > 30%. The poor outcomes observed in patients harbouring AR aberrations are consistent with previous reports of primary resistance to AA+P^23^. Interestingly, a majority of the patients with detected T878A mutation had a PSA decline following switch, although median bPFS and rPFS were shorter than *AR* normal patients. T878A mutation is activated by 21-carbon-steroids, such as progesterone^24^, and its levels are increased by abiraterone^5^ but suppressed by continuous low-dose dexamethasone^25^. Confirmation of our results in additional cohorts would allow the selection of patients likely to benefit from the steroid switch^14,23^. We have also analysed *ERG* rearrangements and *PTEN* expression status suggested as potential biomarkers of AA + P benefit^12,26^. However, in our small series the absence of *ERG* rearrangements seemed related to PSA responses but not to rPFS.

We acknowledge that our study has some limitations. First, the lack of a control arm makes it impossible to establish the exact clinical benefit that can be obtained with a steroid switch, beyond PSA responses; a future randomised phase II study should include a control group in which AA+P is continued until radiographic progression. A second limitation is that molecular analyses did not include other potential predictive biomarkers such as *AR-V7* or the glucocorticoid receptor (GR). Although *AR* splicing variants have been linked to resistance to AA+P^27^, GR has been suggested to lead to the cross-stimulation of AR target genes in the absence of androgens^28^.

## Conclusion

Our study provides prospective evidence that a steroid switch is a feasible and safe manoeuvre that can induce responses in clinically stable patients progressing on abiraterone. We also present hypothesis-generating evidence of the role of *AR* amplifications, *AR* mutations and *ERG* rearrangements as potential predictive biomarkers. Nonetheless, these findings require further validation, ideally in a prospective, randomised clinical trial.

## Electronic supplementary material

Suppl. Apendix
