# Supplementary material for: Phase II pilot study of the prednisone to dexamethasone switch in metastatic castration-resistant prostate cancer (mCRPC) patients with limited progression on abiraterone plus prednisone (SWITCH study)
Source: Br J Cancer. 2018 Aug 21;119(9):1052–9. doi: 10.1038/s41416-018-0123-9 (PMC6219494; doi:10.1038/s41416-018-0123-9)
Supplement: Supplementary file 1 — Suppl. Apendix [file 41416_2018_123_MOESM1_ESM.docx]

**SUPPLEMENTARY APPENDIX**

**S1. Full inclusion an exclusion criteria**

Inclusion criteria

1. The subject is capable of understanding and complying with the protocol requirements and has signed the informed consent document.
2. Age ≥18 years.
3. ECOG Performance Status of ≤ 2.
4. Histologically confirmed adenocarcinoma of the prostate.
5. Documented castration resistance prostate cancer according to PCWG2 criteria, under treatment with LHRH agonists (or orchiectomy) with testosterone levels of < 50ng/dl (< 2.0 nM).
6. Candidates to receive treatment with abiraterone acetate 1000mg/24h + prednisone 10mg od and prednisone 5mg bid.
7. Biochemical progression to abiraterone plus prednisone assessed by the investigator with a PSA value at the screening visit ≥ 2µg/L (ng/ml) and a 25%or greater increase over PSA nadir with a second confirmatory measurement with an interval of ≥ 2 weeks.
8. Asymptomatic or oligosymptomatic patients that maintain clinical stability during treatment with abiraterone plus prednisone, without signs of clinical progression when biochemical progression is documented.
9. Absence of radiographic progression except those cases with limited radiological progression (see exclusion criteria 1).
10. Subjects must have adequate bone marrow, hepatic and renal function documented, without contraindication to administrate abiraterone according to data sheet.

Exclusion criteria

1. Radiological progression: 1a) New nodal or visceral metastasis during treatment with abiraterone plus prednisone or an increase > 40% of target lesions by RECIST criteria 1.1; 1b) More than three new bone metastasis during treatment with abiraterone plus prednisone. Those patients with limited radiological progression (3 or less new bone metastasis or an increase ≤ 40% in target lesions) will be accepted for inclusion, if no clinical progression is associated.
2. Previous cancer diagnosis, except those patients who had a localized malignant tumour and who are five years cancer-free, as well as subjects with a history of skin cancers (of non-melanoma type) or excised in situ carcinomas.
3. Any medical condition that contraindicate to receive dexamethasone as adjuvant therapy of treatment with abiraterone acetate.
4. Medical condition which according to the judgement of the investigator, might interfere with the subject`s granting of informed consent ot the safe execution of the procedures required in the study.

**S2. Biomarkers Studies**

**A) Studies in archived FFPE**

Available archival prostate cancer formalin-fixed paraffin-embedded (FFPE) samples were collected and reviewed by a trained uro-pathologist blinded to patients who marked tumour areas. PTEN protein expression was determined by IHC on 4-mM-thick FFPE sections as previously described[^1^](#_ENREF_15) using a rabbit monoclonal anti-PTEN antibody 138G6 (catalog no. 9559; Cell Signaling Technology, Inc, Danvers, MA, USA) [^1^](#_ENREF_15). *TMPRSS2-ERG* fusion and *ERG* amplification were assessed by fluorescent in-situ hybridisation (FISH) using a three-colour assay based on the ERG break-apart assay described by Attard et al^2, 3^ and adding three additional BAC probes (RP11-282I20, RP11-891L10, RP11-35C40) flanking the 5’ *TMPRSS2* region. Tissues were then scanned using an Ariol SL-50 scanner (Applied Imaging. Tumour areas were independently scored for *TMPRSS2-ERG* by NRL and EC.

**b) AR copy-number analysis in circulating tumour DNA (ctDNA)**

Plasma was obtained by centrifugation of 20 mL of blood collected in EDTA tubes within 2-hours from blood-drawn and stored at -80ºC for later ctDNA isolation. ctDNA was extracted from 1-2 ml of plasma with the QIAamp Circulating Nucleic Acid Kit (Qiagen). Total extracted plasma DNA was quantified with the Quant-iT high sensitivity PicoGreen dsDNA Assay Kit (Invitrogen). Digital drop PCR (ddPCR) was performed on a QX200 ddPCR system (Bio-Rad). Copy number (CN) assays were performed for *AR* (Hs04121925_cn, FAM) and centromeric chromosome X gene *ZXDB* (Hs02220689_cn, FAM, Life Technologies) with *NSUN3* (dHsaCP2506682, HEX, Bio-Rad), *ElF2C1* (dHsaCP1000002, HEX, Bio-Rad), and *AP3B1* (dHsaCP1000001, HEX, Bio-Rad) as reference genes. Rare mutation detection assays were performed for the *AR* mutations 2105T>A (p.L702H), and 2632A>G (p.T878A) using a custom-made single nucleotide polymorphism (SNP) genotyping assay (Life Technologies), and the SNP genotyping assay rs137852578 (Life Technologies), respectively.[^4-6^](#_ENREF_18)

**S3. CONSORT DIAGRAM**

Evaluable plasma for AR status (n=17)

Eligibility pre-assesment

(n= 61 patients)

Enrollment

June 2013 – March 2016

Consented and screened

n=29

A priori not elegible (n=23)

- Symptoms worsening (n=16)
- Radiological progression (n=7)

Declined to participate (n=9)

Evaluable tissue for IHC & FISH (n=17)

Screening Failures (n=3)

- Uncontrolled symptoms (n=1)
- Significant Radiographic progression (n=2)

Enrolled & Elegible

for response and safety

n=26

Archival tissue (n=20)

Blood sample

(n= 19)

Optional biomarkers

n=20

**S4. Baseline characteristics according to pre- or post-docetaxel status**

**S5. SUBSEQUENT THERAPIES AFTER AA+D AND REPONSES**


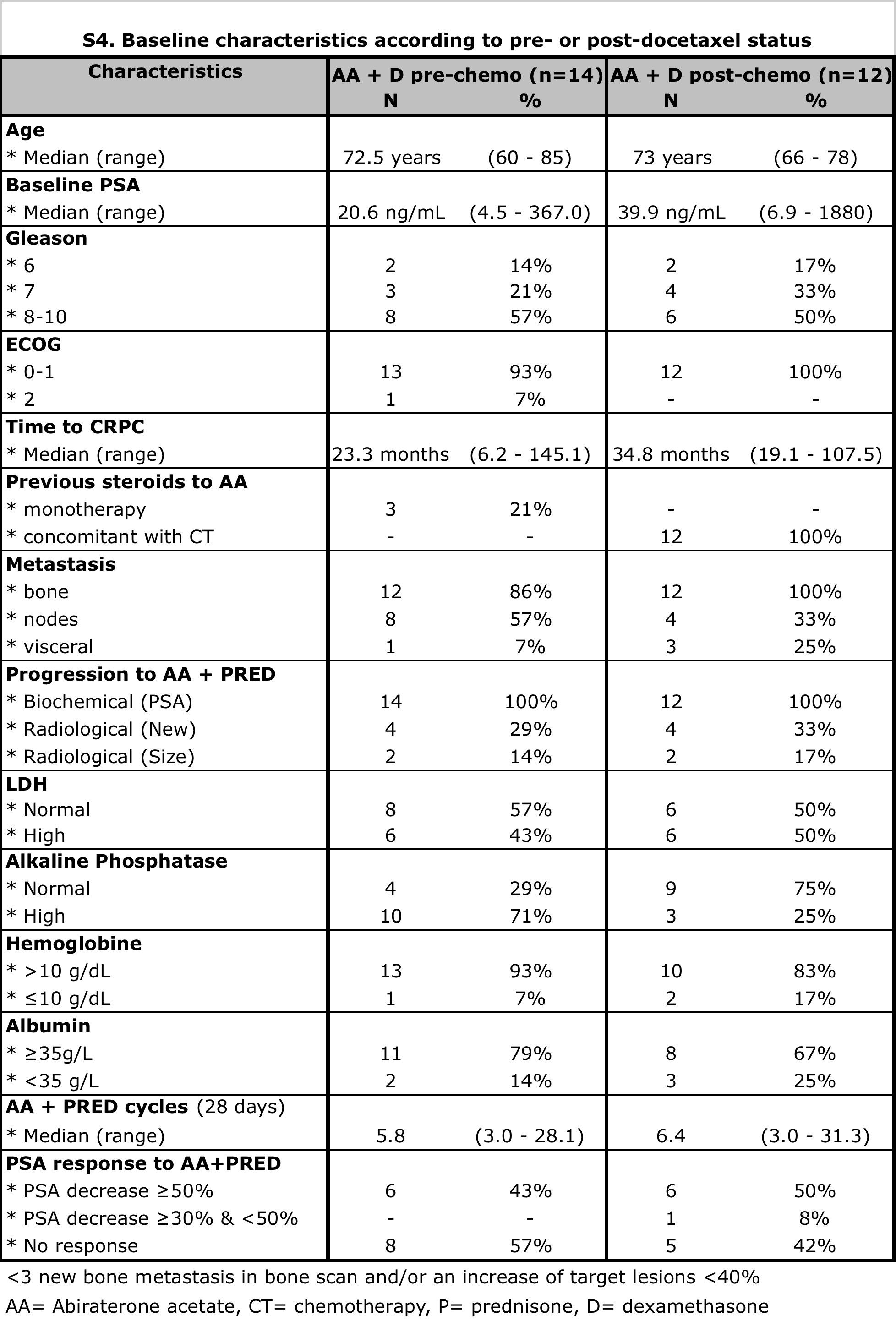


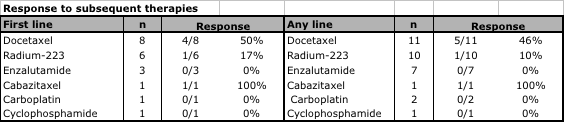


**S6. PLASMA AND ARCHIVED TISSUESAMPLES**


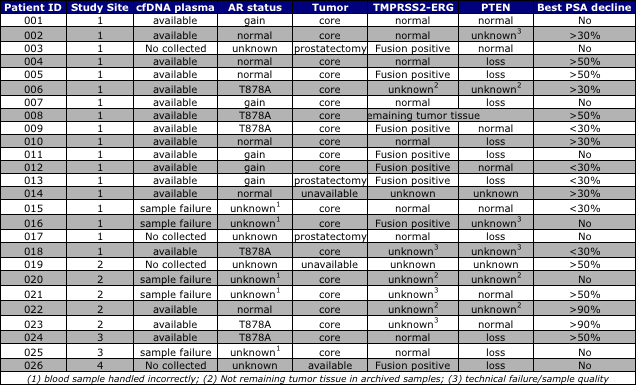


**REFERENCES SUPPLEMENTARY MATERIAL**

**1.** Ferraldeschi R, Nava Rodrigues D, Riisnaes R, et al. PTEN protein loss and clinical outcome from castration-resistant prostate cancer treated with abiraterone acetate. *European urology.* Apr 2015;67(4):795-802.

**2.** Clark J, Attard G, Jhavar S, et al. Complex patterns of ETS gene alteration arise during cancer development in the human prostate. *Oncogene.* Mar 27 2008;27(14):1993-2003.

**3.** Attard G, Clark J, Ambroisine L, et al. Duplication of the fusion of TMPRSS2 to ERG sequences identifies fatal human prostate cancer. *Oncogene.* Jan 10 2008;27(3):253-263.

**4.** Taly V, Pekin D, Benhaim L, et al. Multiplex picodroplet digital PCR to detect KRAS mutations in circulating DNA from the plasma of colorectal cancer patients. *Clinical chemistry.* Dec 2013;59(12):1722-1731.

**5.** Garcia-Murillas I, Schiavon G, Weigelt B, et al. Mutation tracking in circulating tumor DNA predicts relapse in early breast cancer. *Science translational medicine.* Aug 26 2015;7(302):302ra133.

**6.** Gevensleben H, Garcia-Murillas I, Graeser MK, et al. Noninvasive detection of HER2 amplification with plasma DNA digital PCR. *Clinical cancer research : an official journal of the American Association for Cancer Research.* Jun 15 2013;19(12):3276-3284.
